# Unleashing the full potential of Hsp90 inhibitors as cancer therapeutics through simultaneous inactivation of Hsp90, Grp94, and TRAP1

**DOI:** 10.1038/s12276-019-0360-x

**Published:** 2020-01-20

**Authors:** Hye-Kyung Park, Nam Gu Yoon, Ji-Eun Lee, Sung Hu, Sora Yoon, So Yeon Kim, Jun-Hee Hong, Dougu Nam, Young Chan Chae, Jong Bae Park, Byoung Heon Kang

**Affiliations:** 1 Department of Biological Sciences, Ulsan National Institutes of Science and Technology (UNIST), Ulsan, 44919 South Korea; 2 Rare Cancer Branch, Research Institute and Hospital, National Cancer Center, Goyang, 10408 Republic of Korea; 3 Department of System Cancer Science, Graduate School of Cancer Science and Policy, National Cancer Center, Goyang, Korea

**Keywords:** Drug development, Chaperones

## Abstract

The Hsp90 family proteins Hsp90, Grp94, and TRAP1 are present in the cell cytoplasm, endoplasmic reticulum, and mitochondria, respectively; all play important roles in tumorigenesis by regulating protein homeostasis in response to stress. Thus, simultaneous inhibition of all Hsp90 paralogs is a reasonable strategy for cancer therapy. However, since the existing pan-Hsp90 inhibitor does not accumulate in mitochondria, the potential anticancer activity of pan-Hsp90 inhibition has not yet been fully examined in vivo. Analysis of The Cancer Genome Atlas database revealed that all Hsp90 paralogs were upregulated in prostate cancer. Inactivation of all Hsp90 paralogs induced mitochondrial dysfunction, increased cytosolic calcium, and activated calcineurin. Active calcineurin blocked prosurvival heat shock responses upon Hsp90 inhibition by preventing nuclear translocation of HSF1. The purine scaffold derivative DN401 inhibited all Hsp90 paralogs simultaneously and showed stronger anticancer activity than other Hsp90 inhibitors. Pan-Hsp90 inhibition increased cytotoxicity and suppressed mechanisms that protect cancer cells, suggesting that it is a feasible strategy for the development of potent anticancer drugs. The mitochondria-permeable drug DN401 is a newly identified in vivo pan-Hsp90 inhibitor with potent anticancer activity.

## Cancer therapeutics: Extending a drug’s reach

A new drug that blocks heat shock proteins (HSPs), helper proteins that are co-opted by cancer cells to promote tumor growth, shows promise for cancer treatment. Several drugs have targeted HSPs, since cancer cells are known to hijack these helper proteins to shield themselves from destruction by the body. However, the drugs have had limited success. Hye-Kyung Park and Byoung Heon Kang at Ulsan National Institutes of Science and Technology in South Korea and coworkers noticed that the drugs were not absorbed into mitochondria, a key cellular compartment, and HSPs in this compartment were therefore not being blocked. They identified a new HSP inhibitor that can reach every cellular compartment and inhibit all HSPs. Testing in mice showed that this inhibitor effectively triggered death of tumor cells, and therefore shows promise for anti-cancer therapy.

## Introduction

Heat shock protein 90 (Hsp90) family members are ATP-dependent molecular chaperones that regulate the stability and function of client proteins involved in growth, survival, and adaptation of cancer cells to cellular stress^1–3^. Hsp90 family proteins comprise four paralogs, each of which resides at different subcellular locations: Hsp90α/β in the cytoplasm, glucose-regulated protein 94 (Grp94) in the endoplasmic reticulum (ER), and tumor necrosis factor receptor-associated protein-1 (TRAP1) in mitochondria. Each Hsp90 paralog plays a crucial role in tumor progression, multidrug resistance, increased cell death threshold, and metastasis^4–8^. Thus, simultaneous inactivation of all Hsp90 family proteins can compromise multiple tumorigenic pathways operating in different cellular compartments; this approach is very likely to increase anticancer activity above that afforded by paralog-specific inactivation of Hsp90 family proteins.

Overall, Hsp90 paralogs have a similar structure and very high similarity with respect to the ATP binding pockets at the N-terminal domains (NTDs)^9,10^. Therefore, many Hsp90 inhibitors targeting the ATP binding pocket exhibited cross-reactivity against both Grp94 and TRAP1 in vitro; such agents are known as pan-Hsp90 inhibitors^11^. Recently, however, we demonstrated that current pan-Hsp90 inhibitors do not inactivate TRAP1 significantly in vivo due to inefficient accumulation of the drug inside mitochondria^12,13^. Thus, the reported cellular activity of most Hsp90 inhibitors originates primarily from inactivation of cytosolic and/or ER Hsp90 paralogs, not from inactivation of mitochondrial TRAP1. Thus, no study has yet examined the anticancer activity of actual pan-Hsp90 family protein inhibitors, meaning that the therapeutic potential/benefits of simultaneous inactivation of all Hsp90 family proteins have never been explored.

Since Hsp90 was identified as a potential cancer target, various inhibitors targeting cytoplasmic Hsp90 have been developed as anticancer agents^1–3,14^. So far, at least 18 different inhibitors with desirable pharmacological properties, including ganetespib (STA-9090), have been evaluated in clinical trials, but none have shown satisfactory efficacy to be approved by the FDA. The main reason for the limited efficacy can be attributed to the dose-limiting toxicity at the drug dose of near-complete client depletion and the inevitable activation of HSF1, leading to cytoprotective heat shock responses^15–17^.

HSF1 is a transcription factor that causes heat shock responses such as induction of Hsp27, Hsp40, and Hsp70 to allow cells to adapt to proteotoxic stresses^18,19^. These target genes of HSF1 have powerful cytoprotective functions, including anti-apoptosis, drug resistance, and proliferative effects^20^. Upon proteotoxic stress, Hsp90 binds to unfolded client proteins and dissociates HSF1 from HSP90, which in turn induces translocation of HSF1 to the nucleus and subsequent expression of heat shock response genes. Similarly, Hsp90 inhibitors trigger dissociation of HSF1 from Hsp90 and activate the prosurvival heat shock response, which is a mechanical drawback and an unavoidable adverse effect of current Hsp90 inhibitors^19,21,22^. Thus, coinhibition of HSF1 has been suggested to improve the anticancer activity of Hsp90 inhibitors^23,24^.

Calcineurin is a calcium-dependent serine/threonine protein phosphatase whose activation inhibits that of HSF1^25–27^. Activated calcineurin dephosphorylates and subsequently suppresses nuclear translocation of HSF1. In addition to its antitumorigenic roles, calcineurin supports protumorigenic pathways by activating nuclear factor of activated T cells (NFAT). Critically, activation and nuclear translocation of NFAT is regulated by the phosphorylation status of the transcription factor^28^. Among Hsp90 client proteins, Akt inactivates glycogen synthase kinase 3β (GSK3β), which triggers NFAT export to suppress its transcriptional activity. Thus, complex regulatory networks involving protumorigenic transcription factors HSF1 and NFAT would be markedly affected by dysregulation of calcium homeostasis and Hsp90.

Here, we show that simultaneous inactivation of Hsp90 paralogs localized within different cellular compartments not only increases anticancer activity but also dysregulates calcium homeostasis. In addition, activation of HSF1 and NFAT was suppressed by dysregulation of calcium and Hsp90 homeostasis, which further increases the anticancer activity of Hsp90 inhibitors.

## Materials and methods

### Cells and culture conditions

Human cervical (HeLa), liver (SK-HEP-1, HepG2), brain (A172), kidney (ACHN), lung (H460), and prostate (22Rv1) cancer cells were purchased from the American Type Culture Collection (ATCC) and maintained as recommended by the supplier. Cells were cultured at 37 °C in a humidified atmosphere of 5% CO_2_ and in DMEM or RPMI medium (GIBCO) containing 10% fetal bovine serum (FBS; GIBCO) and 1% penicillin/streptomycin (GIBCO). Human RWPE-1 and corneal cells were purchased from ATCC and cultured in ATCC corneal medium (ATCC). Primary hepatocytes were isolated from 8-week-old BALB/c mice, as described previously^13^{Park, 2014 #17}{Park, 2014 #17} and cultured in M199/EBSS medium (HyClone) containing 10% FBS at 37 °C/5% CO_2_.

### Chemicals and antibodies

All chemicals were purchased from Sigma. Anti-TRAP1, anti-Hsp90, anti-Cyt c, anti-Hsp70, anti-Drp1, and anti-Bax antibodies were purchased from BD Biosciences; anti-Grp94, anti-Akt, anti-Chk1, anti-c-FLIP, anti-HER2, anti-Sorcin, and anti-SDHB antibodies were purchased from Santa Cruz Biotechnology; anti-PHB, anti-pS9-GSK3β, anti-GSK3β, anti-COX-2, anti-HSF1, anti-SIRT3, and anti-calcineurin A antibodies were purchased from Cell Signaling Technology; an anti-calcineurin regulatory subunit B antibody was purchased from Sigma; an anti-CypD antibody was purchased from Thermo; and an anti-β-actin antibody was purchased from MP Biomedicals.

### Cellular calcineurin activity assays

Cellular calcineurin activity was measured using a calcineurin phosphatase assay kit (ENZO Life Sciences). Colorimetric assays were performed at a wavelength of 620 nm using a microplate spectrophotometer (TECHAN Infinite M200). Absorbance values were normalized to protein concentrations, and calcineurin activity was expressed relative to that in DMSO-treated samples.

### siRNA treatment

Small interfering RNAs (siRNA) specific for calcineurin, ryanodine receptor 2 (RyR2), cyclophilin D (CypD), TRAP1, and Grp94 were synthesized by Genolution (Korea). siRNAs targeting CypD and calcineurin regulatory subunit B (CNB) were synthesized by Genolution (Korea) using the following sequences: CNB-#1 5′-GCCTGAGTTACAACAGAATCCTTTA -3′ and CNB-#2 5′-GGAACAATCTGAAAGATACACAGTT-3′; RyR2-#1 5′-AAGTGGTTCTGCAGTGCACCG; RyR2-#2 5′-AAGTACGAGTTGGAGATGACC; CypD-#1 5′-GGCAGATGTCGT-CCCAAAG-3′ and CypD-#2 5′-GATAAGGGCTTCGGCTACA-3′; Grp94-#1 5′- TCGCCTCAGTTTGAACATTGA and Grp94-#2 5′- GAGAGAGGAAGAAGCTATT; TRAP1-#1 5′-AAACATGAGTTCCAGGCCGAG and TRAP1-#2 5′-CCCGGTCCCTGTACTCAGAAA; and control 5′-ACUCUAUCUGCACGCUGAC-3′. Cells were cultured on six-well plates until they reached 50–75% confluence, transfected for 48 h with 20 nM siRNA mixed with G-Fectin (Genolution), and then treated with drugs as indicated.

### Protein purification and fluorescence polarization assays

Recombinant full-length TRAP1, Hsp90α, and GST-Grp94 were prepared as described previously^13^. During preparation, the recombinant GST-Grp94 protein was eluted with a buffer containing 10 mM glutathione as the last step. The fluorescence probe PU-H71-FITC3 was synthesized as described previously and used to measure fluorescence polarization^13^. Briefly, 10 nM PU-H71-FITC3 and 100 nM protein were incubated for 24 h at 4 °C with various concentrations of inhibitor in FP buffer containing 135 mM NaCl, 4.3 mM Na_2_HPO_4_, 2.7 mM KCl, 1.4 mM KH_2_PO_4_, 2 mM MgCl_2_, 1 mM DTT, 0.1 mg/mL BSA, and 0.05% NP40 (pH 7.3). Fluorescence polarization was measured in a SYNERGY NEO microplate reader (BioTek Instruments, Inc.).

### Analyses of cell viability and apoptosis induction

Cell viability was examined using 3(4,5-dimethyl-thyzoyl-2-yl)2,5 diphenyltetrazolium bromide (MTT) assays and quantified by measuring the absorbance of the tetrazolium dye at 595 nm in a SYNERGY NEO microplate reader (BioTek Instruments, Inc.). Absorbance values were normalized to those of the DMSO control, and the data were expressed as percentage viability. To measure apoptosis induction, caspase activation (DEVDase activity) and the DNA content (propidium iodide) of labeled cells were analyzed using a FACSCalibur™ flow cytometer (BD Biosciences). Data were processed using FlowJo software (TreeStar). In situ direct DNA fragmentation was analyzed using an in situ cell death detection kit (Roche) and an FV1000 laser confocal scanning microscope (Olympus).

### Live cell imaging

Cytoplasmic calcium concentrations were determined as previously described^29^. Briefly, HeLa cells were labeled for 30 min with 5 μM Fura-2 and incubated at 37 °C/5% CO_2_ in calcium-free Locke’s solution containing 154 mM NaCl, 5.6 mM KCl, 3.2 mM MgCl_2_, 5 mM HEPES, 10 mM glucose, and 0.2 mM EGTA (pH 7.4). After drug treatment, changes in fluorescence were monitored every 5 min using an IX81 ZDC microscope (Olympus) at an emission wavelength of 510 nm, with dual excitation at 340 and 380 nm. Images of 340/380 fluorescence ratios were generated and analyzed using the Xcellence software package (Olympus). To detect HSF1 localization, HeLa cells were transfected with an HSF1–GFP plasmid^30^ obtained from Addgene (Addgene plasmid #32538) using the Lipofectamine transfection reagent (Invitrogen). After transfection for 24 h, cells were treated with the indicated drugs and then analyzed using an IX81 ZDC (Olympus). To visualize mitochondrial structure, HeLa cells were stained for 20 min with 200 nM MitoTracker dye, treated with the drugs, and then analyzed under an LSM 780 confocal microscope (Zeiss).

### Immunohistofluorescence staining of prostate tumor specimens

Human specimens were provided by the Korea Prostate Bank through the Infrastructure Project for Basic Science of the MEST, Korea. Experiments using tumor tissues from patients with prostate cancer (PCa) were approved by the Institutional Review Board (IRB) of UNIST (IRB no. 1044216-13-07-C). Formalin-fixed, paraffin-embedded tissue samples from patients were obtained from the Korea Prostate Bank (Seoul, Korea). Sections were deparaffinized by incubation at 60 °C for 2 h and then rehydrated through a series of graded ethanol solutions, culminating in water alone. Antigen retrieval was performed in 10 mM sodium citrate (pH 6.0) for 40 min using a conventional pressure cooker. Cells were permeabilized with 0.5% Triton X-100 for 10 min and then blocked with blocking solution (5% BSA, 5% FBS, 0.3% Triton X-100) for 1 h at room temperature. Dual-immunostaining was performed using anti-mouse Hsp90/anti-rabbit TRAP1 and anti-mouse TRAP1/anti-rabbit Grp94 antibodies, followed by Alexa-488 and Alexa-594 conjugated secondary antibodies. Stained tissues were analyzed using an Axio zoom microscope (Zeiss).

### RNA extraction and reverse transcription-polymerase chain reaction (RT-PCR)

Total RNA was prepared from cultured cells using RNeasy mini kits (QIAGEN), and cDNA was synthesized using the ProtoScript® First Strand cDNA Synthesis Kit (New England Biolabs) and an oligo (dT) primer. PCR reactions were performed in a Master cycler PCR machine (Eppendorf) with the following sets of oligonucleotide primers: GAPDH, 5′-CGGGAAGCTTGTCATCAATGG-3′ and 5′-GGCAGTGATGGCATGGACTG-3′; Hsp27, 5′-GAGTGGTCGCAGTGGTTAGG-3′ and 5′-ACAGGGAGGAGGAAACTTGG-3′; Hsp40, 5′-GGAAAGAGCATTCGAAACGA-3′ and 5′-ATGCCAGGCCTGATAACATC-3′; Hsp70, 5′-CCAGCTGAAGAAGGGTCAAG-3′ and 5′-TTTTCTGCTGGTGTCTGCTG-3′; and Hsp90, 5′-CTGGGTTTCCTCAGGAT-3′ and 5′-TACCGGATTTTGTCCAATGC-3′.

### Tumor xenograft experiments

All experiments involving animals were approved by the Ulsan National Institute of Science and Technology (UNIST, UNISTIACUC-16-27). Cancer cells (7 × 10^6^ 22Rv1) were suspended in 200 μL of PBS and then injected subcutaneously into both flanks of 6-week-old BALB/c nu/nu male mice (Charles River Laboratories). Tumors were allowed to grow to an average volume of approximately 100 mm^3^. Next, animals were grouped randomly (two tumors/mouse, five mice/group). Subsequently, vehicle (DMSO), DMAG, and gamitrinib were dissolved in 20% Cremophor EL (Sigma) in PBS. DMAG and/or gamitrinib (10 mg/kg) were administered intraperitoneally twice a week. Tumors were measured using calipers, and tumor volumes were calculated using the following formula: *V* = 1/2 × (width)^2^ × length. At the end of the experiment, animals were euthanized, and organs (brain, heart, kidney, liver, lung, spleen, stomach, and tumor) were collected for histology and western blot analysis. In addition, harvested organ specimens were fixed in 10% formalin and embedded in paraffin for histological analyses. Briefly, Section (5 μm thick) were placed on high-adhesive slides, stained with H&E, and scanned at ×20 magnification using the Dot slide system (Olympus). For western blot analyses, tissue samples were homogenized in RIPA buffer containing 50 mM Tris (pH 8.0), 150 mM NaCl, 1% NP-40, and 0.25% *N*-deoxycholate, plus protease inhibitor and phosphatase inhibitor cocktails (Calbiochem).

### Data from The Cancer Genome Atlas (TCGA)

The Cancer Genome Atlas (TCGA) level 3 PCa RNAseq data were downloaded from the Broad Institute GDAC FireBrowse (http://firebrowse.org, data version: 2016_01_28). Data were quantified at the gene level using RSEM and normalized using the Upper Quartile method, RPKM (reads per million of mapped reads per kilobase of transcript length), and the *ACTB* gene. Among 550 samples obtained, 52 pairs of cancer and matched normal samples were used for analysis.

### Statistical analyses

MTT experiments were conducted in duplicate and repeated independently at least three times. Statistical analyses were performed using the software program Prism 7.0 (GraphPad). Differences were identified using unpaired *t*-tests and were considered significant at *p* < 0.05.

## Results

### Increased expression of Hsp90, Grp94, and TRAP1 in human PCa specimens

**Fig. 1:**
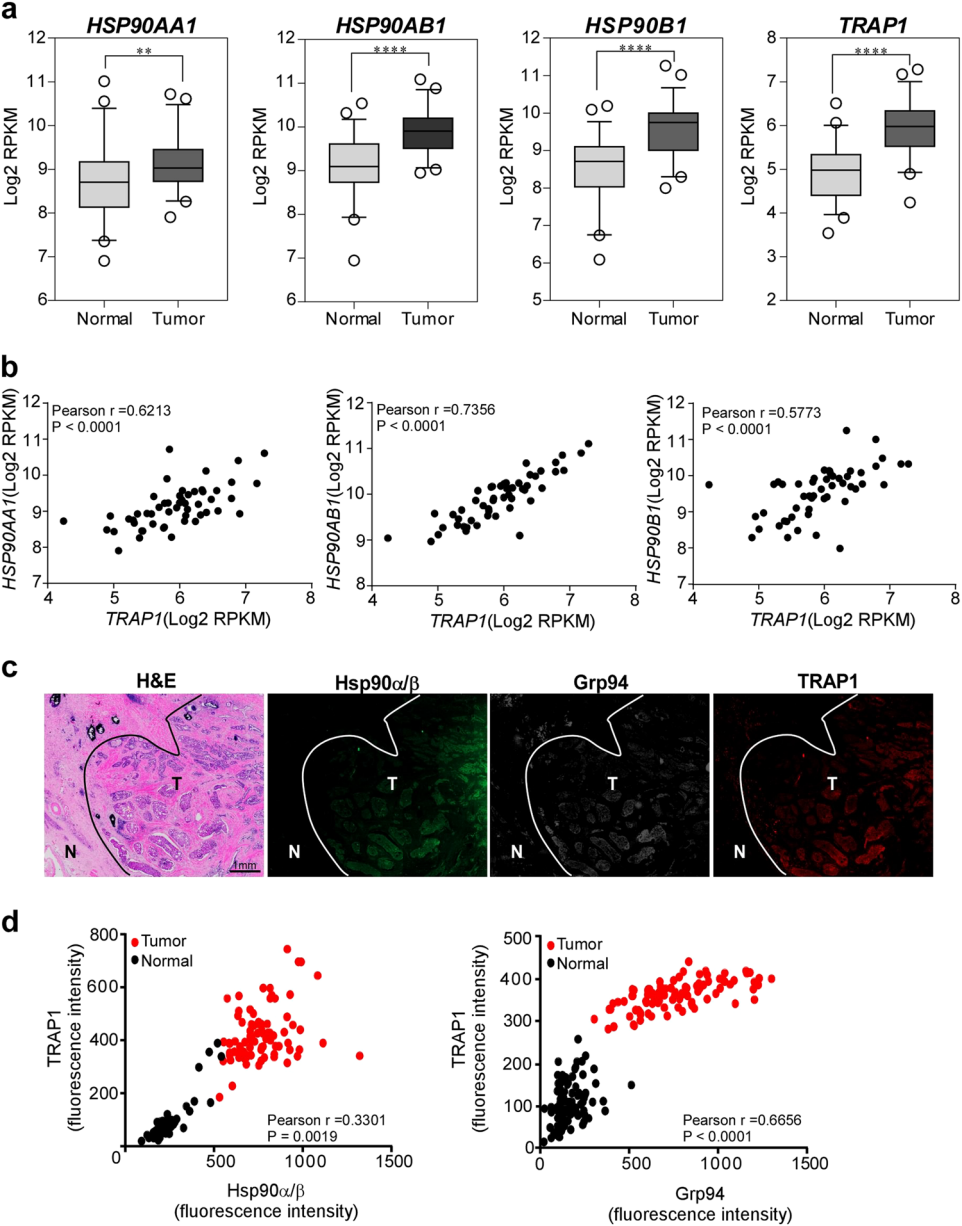
Hsp90 paralogs are coexpressed in prostate tumors. **a** Expression of mRNA encoding Hsp90 gene family members obtained from TCGA RNAseq data. Expression of mRNA encoding *HSP90AA1, HSP90AB1* (Hsp90α/β), *HSP90B1* (Grp94), and *TRAP1* (TRAP1) in cancer and normal tissues from 52 patients with prostate cancer; **p < 0.005, ****p < 0.0001. **b** Correlation between *HSP90AA1* and *TRAP1* (left), *HSP90AB1* and *TRAP1* (middle), and *HSP90B1* and *TRAP1* (right) expression in the TCGA RNAseq database. **c** Expression of Hsp90 paralogs in human prostate cancer specimens. The boundary between the normal (N) and tumor (T) regions is indicated. Tumor specimens were analyzed by immunofluorescence staining with anti-TRAP1, anti-Hsp90, and anti-Grp94 antibodies, and protein expression in single cells was analyzed by confocal microscopy. Scale bar, 1 mm. **d** Expression of TRAP1 vs. Hsp90 (left) and TRAP1 vs. Grp94 (right). Tumor specimens were analyzed as in **c**. Data from 87 cells are presented in scatter plots. Pearson correlation coefficient (*r*) and *p* values are indicated.

We analyzed PCa RNAseq data from The Cancer Genome Atlas (TCGA) to examine the expression of Hsp90α (*HSP90AA1*), Hsp90β (*HSP90AB1*), Grp94 (*HSP90B1*), and TRAP1 (*TRAP1*). The expression of genes in tumors was significantly higher than that in normal tissues (Fig. 1a). Although some Hsp90 inhibitors inhibit all Hsp90 family proteins in vitro, they are unable to inhibit mitochondrial Hsp90 in vivo due to inefficient accumulation inside mitochondria^13,31^. Thus, we analyzed the pairwise expression of Hsp90 paralogs between Hsp90 inhibitor-inhibited Hsp90/Grp94 and noninhibited TRAP1. Pairwise comparison revealed a strong positive correlation between TRAP1 and other Hsp90 paralogs; Spearman’s correlation coefficients (*r*) for Hsp90α, Hsp90β, and Grp94 were 0.6213, 0.7356, and 0.5773, respectively (Fig. 1b). Consistent with these data, tumor regions in human PCa specimens showed higher expression of Hsp90(α/β), Grp94, and TRAP1 proteins than normal surrounding tissues (Fig. 1c, d). Further analyses of the confocal fluorescence microscopy images revealed a significant positive correlation between TRAP1 expression and that of Hsp90 (*r* = 0.3301) and Grp94 (*r* = 0.6656) at a single cancer cell level (Fig. 1d). Taken together, these data indicate increased expression of all Hsp90 paralogs in tumors and suggest that inactivation of all Hsp90 paralogs could increase anticancer activity.

### Combination treatment with TRAP1 and Hsp90 inhibitors induces apoptosis in vitro and in vivo

**Fig. 2:**
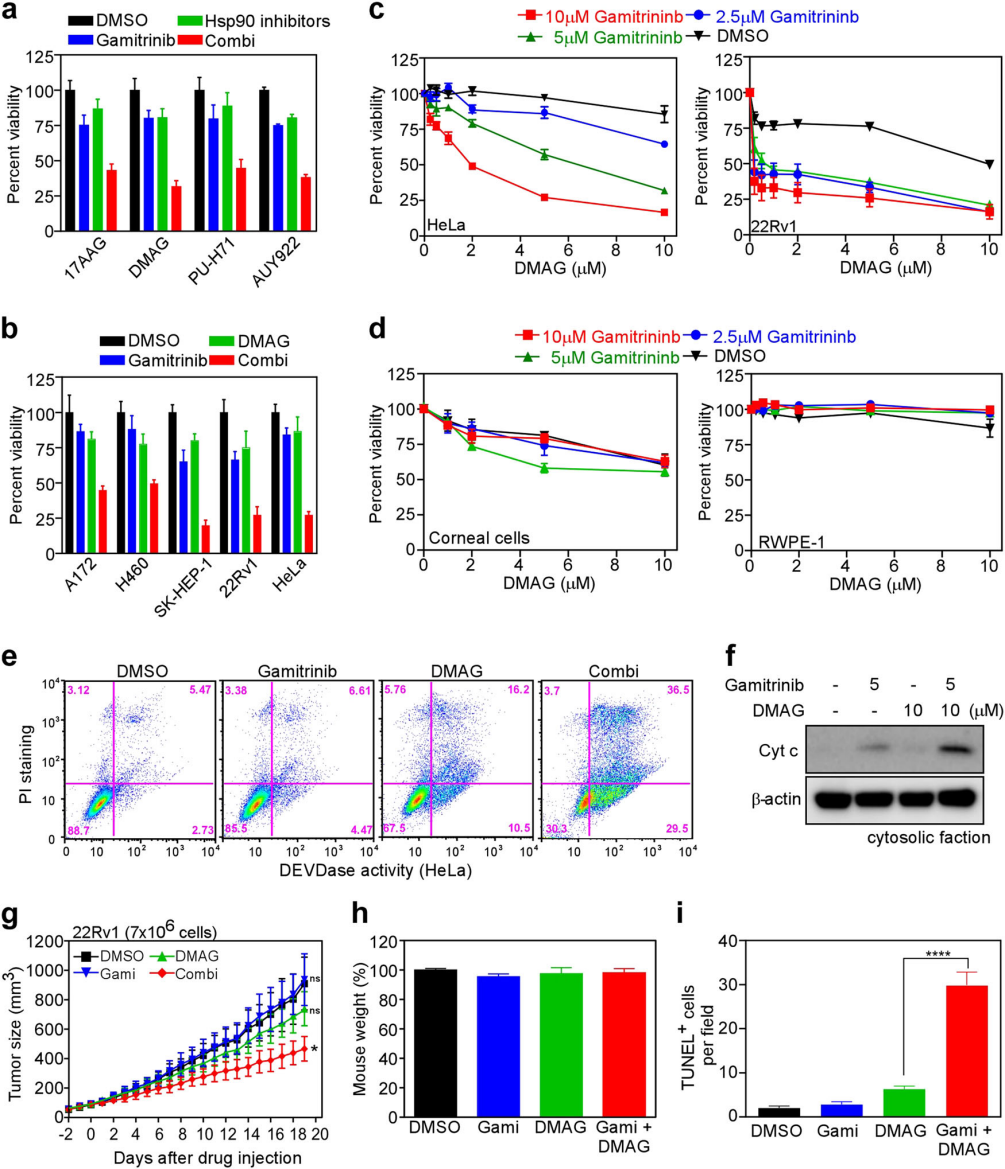
Synergistic anticancer effects of combined treatment with gamitrinib and Hsp90 inhibitors. **a** Combined treatment with Hsp90 inhibitors plus gamitrinib. HeLa cells were treated with 5 μM gamitrinib and 10 μM Hsp90 inhibitors for 24 h and then analyzed by the MTT assay. **b** Effect of combined drug treatment on various cancer cell lines. 22Rv1 cells were treated for 24 h with 2.5 μM gamitrinib and 5 μM DAMG, and other cells were treated with 5 μM gamitrinib and 10 μM DMAG, either alone or in combination, and then analyzed by the MTT assay. **c** Synergistic cytotoxic activity. HeLa and 22Rv1 cells were treated with various concentrations of DMAG in the presence of 2.5, 5, and 10 μM gamitrinib and then analyzed by the MTT assay. **d** Cytotoxicity against human normal cells. Primary human corneal cells and normal human prostate normal cells (RWPE-1) were treated for 24 h with drugs and then analyzed by the MTT assay. **e** Induction of apoptosis. HeLa cells were treated for 24 h with 5 μM gamitrinib and 10 μM DMAG, either alone or in combination, stained with propidium iodide (PI) and FITC-DEVD-fmk, and then analyzed by flow cytometry. **f** Cytochrome c (Cyt C) discharge from mitochondria. HeLa cells were treated for 18 h with drugs, and cytosolic fractions were analyzed by western blotting. **g** Tumor xenograft experiment. 22Rv1 cells were implanted subcutaneously into nude mice. Mice received 10 mg/kg DMAG (i.p.) and 10 mg/kg gamitrinib (i.p.), either alone or in combination, twice/week. Tumor volume was assessed daily by caliper measurement. Three mice per group/two tumors per animal. **h** Body weight changes were measured prior to sacrifice. **i** Apoptosis of tumor tissues was analyzed by the TUNEL assay. All data represent the mean ± SEM; *p < 0.05, ****p < 0.0001.

To examine the effect of simultaneous inactivation of all Hsp90 paralogs in cancer cells, we treated HeLa cells with Hsp90 inhibitors (to inactivate Hsp90s localized in the cytoplasm and ER) and gamitrinib (to inactivate the mitochondrial pool of Hsp90s, including TRAP1)^31,32^. All Hsp90 inhibitors showed increased cytotoxic effects when combined with gamitrinib (Fig. 2a). This increased cytotoxicity of the drug combination was confirmed in A172, NCI-H460, SK-HEP-1, 22Rv1, and HeLa cells (brain, lung, liver, prostate, and cervical cell lines, respectively) (Fig. 2b). Mathematical analysis using combination index (CI) values^33^ showed that the effect of the drug combination was synergistic, i.e., CI values in cancer cells were > 0.75 (Fig. 2c and Supplementary Table 1). However, drug synergism was not detected when used to treat normal prostate epithelial cells (RWPE-1) and human corneal cells (Fig. 2d). Combined drug treatment resulted in marked elevation of active caspase-3 (Fig. 2e) and discharge of mitochondrial cytochrome c (Cyt c) (Fig. 2f), suggesting a synergistic increase in apoptosis induction. Similarly, a pan-caspase inhibitor (z-VAD-fmk) led to a marked reduction in cytotoxicity induced by the drug combination (Supplementary Fig. 1). Consistent with in vitro experiments, drug combinations also suppressed the growth of 22Rv1 cells implanted subcutaneously into nude mice to a greater extent than single agent treatments (Fig. 2g); no significant weight loss (Fig. 2h) or organ toxicity was observed (Supplementary Fig. 2a). In addition, combined drug administration led to a marked increase in the number of TUNEL^+^ apoptotic cells in the 22Rvl mouse xenograft model (Fig. 2i; Supplementary Fig. 2b) when compared with that in the control.

### Combined drug treatment alters the phosphorylation of HSF1

**Fig. 3:**
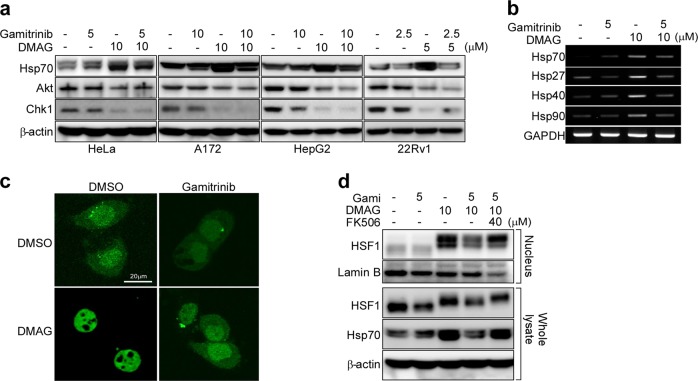
Combined drug treatment inhibits the nuclear localization of HSF1 and suppresses the induction of Hsp70. **a** Suppression of Hsp70 in cancer cells by combined drug treatment. HeLa, A172, HepG2, and 22Rv1 cells were treated for 6 h with gamitrinib and DMAG and then analyzed by western blotting. **b** Expression of HSF1 target genes. 22Rv1 cells were treated for 6 h with gamitrinib and DMAG, and total RNA was isolated and analyzed by reverse transcription PCR. **c** Nuclear translocation of HSF1. HeLa cells showing transient expression of HSF1-GFP were treated for 6 h with 5 μM gamitrinib and 10 μM DMAG, either alone or in combination, and then analyzed by confocal microscopy. Bar, 20 µm. **d** Hyperphosphorylation of HSF1. HeLa cells were treated for 6 h with gamitrinib, DMAG, and FK506 as indicated. The nuclear fraction (upper) and whole cellular extracts (lower) were analyzed by western blotting.

To better understand the molecular mechanisms underlying drug synergism, we first analyzed client protein degradation and induction of heat shock responses in cancer cells after paralog-specific inactivation^18^. As reported previously, treatment with gamitrinib or siRNA targeting TRAP1 degraded its client proteins (SDHB, SIRT3, and Sorcin), and treatment with an Hsp90/Grp94 inhibitor, DMAG, induced degradation of the respective client proteins (Akt, Chk1 and HER2) (Supplementary Fig. 3a, b)^7,9,34–36^. HER2 is a client protein of both Grp94 and Hsp90; thus, treatment with PU-WS13^7^ or siRNA targeting Grp94 induced specific degradation of HER2 (Supplementary Fig. 3a, c). DMAG increased Hsp70 expression in addition to the induction of client proteins, whereas gamitrinib had no effect (Fig. 3a). All data were consistent with those in a previous report of the effects of paralog-specific inhibition in cancer cells^19^. Interestingly, however, DMAG did not increase the expression of heat shock proteins such as Hsp70, Hsp27, Hsp40, and Hsp90 in the presence of gamitrinib (Fig. 3a, b), suggesting a lack of Hsp90 inhibitor-triggered heat shock responses after pan-Hsp90 inhibition. The transcription factor HSF1 is hyperphosphorylated prior to translocation from the cytoplasm to the nucleus, resulting in the expression of heat shock proteins upon Hsp90 inhibition^19,24^. DMAG treatment alone not only increased nuclear localization but also retarded migration of HSF1 in SDS-PAGE gels; by contrast, the DMAG–gamitrinib combination increased HSF1 migration to a greater extent than DMAG alone and inhibited nuclear localization of HSF1 (Fig. 3c, d). These data strongly suggest that defective nuclear localization is caused by altered posttranslational modification of HSF1.

### Combination drug treatment activates calcineurin, which suppresses HSF1 activation

**Fig. 4:**
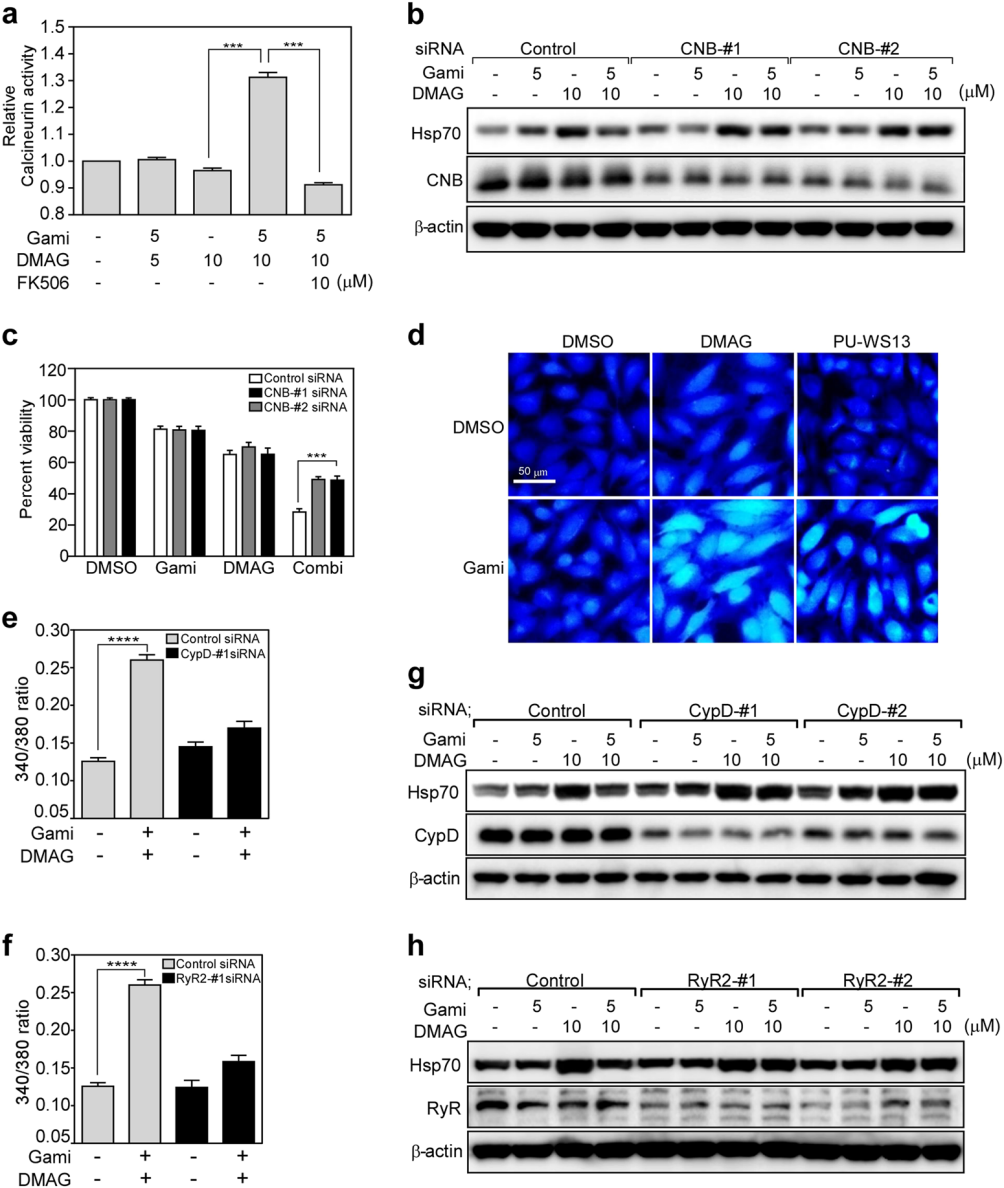
Drug-induced calcium-dependent calcineurin activity promotes cell death. **a** Calcineurin activity. HeLa cells were treated with drugs for 6 h. Calcineurin activity in total cell extracts was analyzed using a calcineurin phosphatase assay kit. Activity was expressed relative to that in DMSO-treated controls. **b** Suppression of Hsp70 by calcineurin silencing. Cells were treated for 24 h with control or calcineurin regulatory subunit (CNB) siRNAs, incubated for 6 h with drugs, and then analyzed by western blotting. **c** Effects of combined drug treatment in the absence of CNB. Cells were treated for 24 h with control or CNB siRNAs, incubated for 24 h with 5 μM gamitrinib and 10 μM DMAG, and then analyzed by the MTT assay. **d** Cytoplasmic calcium release. HeLa cells were stained with Fura-2AM after treatment for 6 h with 5 μM gamitrinib, 10 μM DMAG, and 10 μM PU-WS13 and then analyzed by fluorescence microscopy. Bar, 50 µm. **e** Fura-2AM staining after CypD knockdown. Control or CypD siRNA-transfected cells were treated for 6 h with 5 μM gamitrinib and 10 μM DMAG and then analyzed by fluorescence microscopy. **f** Fura-2AM staining after RyR2 knockdown. Control or RyR2 siRNA-transfected cells were treated for 6 h with 5 μM gamitrinib and 10 μM DMAG and then analyzed by fluorescence microscopy. **g** Hsp70 expression after CypD silencing. After treatment with control or CypD siRNAs, HeLa cells were incubated for 6 h with drugs and then analyzed by western blotting. **h** Hsp70 expression after RyR2 silencing. After treatment with control or RyR2 siRNAs, cells were incubated for 6 h with drugs and analyzed by western blotting. In **a**, **c**, the data are presented as the mean ± SEM; ***p < 0.001. In **e**, **f**, the data are presented as the mean ± SEM of two independent experiments and were collected from 40 cells; ****p < 0.0001.

Since phosphorylation regulates nuclear localization of HSF1^37–40^ and inactivating TRAP1 increases cytoplasmic calcium concentrations^29^, we hypothesized that a calcium-regulated phosphatase could play a role in HSF1 regulation. An inhibitor of the protein phosphatase calcineurin, namely, FK506^41^, reversed the effects of combined drug treatment on HSF1 migration significantly (Fig. 3d), suggesting that calcium and calcineurin regulate HSF1. Consistent with this, combined drug treatment led to a marked increase in calcineurin activity, which was completely inhibited by treatment with FK506 (Fig. 4a). Genetic knockdown of endogenous calcineurin restored the drug combination-induced reduction in Hsp70 expression (Fig. 4b) and significantly reduced the cytotoxicity of the drugs against cancer cells (Fig. 4c). There was no change in the expression of calcineurin protein after combined drug treatment, suggesting altered phosphatase activity of calcineurin (Supplementary Fig. 4a). The data indicate that combined drug treatment-induced cytotoxicity against cancer cells is dependent on the function of calcium-activated calcineurin.

### Inhibition of TRAP1 and Grp94 causes a synergistic increase in calcium release from mitochondria and the ER, respectively

Previous studies report that Grp94 and TRAP1 play roles in calcium homeostasis in the ER and mitochondria, respectively, which are the major calcium-storing organelles^29,42,43^. Therefore, to examine the effect of inhibiting Grp94 and TRAP1 on cytoplasmic calcium elevation, we exclusively inhibited Grp94 using the specific inhibitor PU-WS13^7^ or Grp94-targeting siRNAs. In combination with gamitrinib treatment, inhibition of Grp94 was sufficient to elevate cytoplasmic calcium concentrations (Fig. 4d; Supplementary Fig. 4b, c), suggesting that cancer cells require the chaperone activities of Grp94 and TRAP1 to maintain cellular calcium homeostasis by preserving the proteostasis of respective organelles. Previously, we showed that TRAP1 inactivation increased cytoplasmic calcium concentrations via calcium-induced calcium release mechanisms involving the mitochondria and ER calcium transporters mitochondrial cyclophilin D (CypD) and ER RyR2, respectively^29^. Consistent with this, we found that increases in cytoplasmic calcium in HeLa cells were markedly reduced after genetic knockdown of either CypD or RyR2 (Fig. 4e, f). Furthermore, deficiency of either CypD or RyR2 reversed Hsp70 expression induced by combined drug treatment (Fig. 4g, h), indicating that coordinated calcium discharge between the mitochondria and ER is crucial for suppression of HSF1-dependent heat shock responses mediated by calcineurin activity.

### Akt inhibition blocks calcineurin-induced NFAT survival signals

**Fig. 5:**
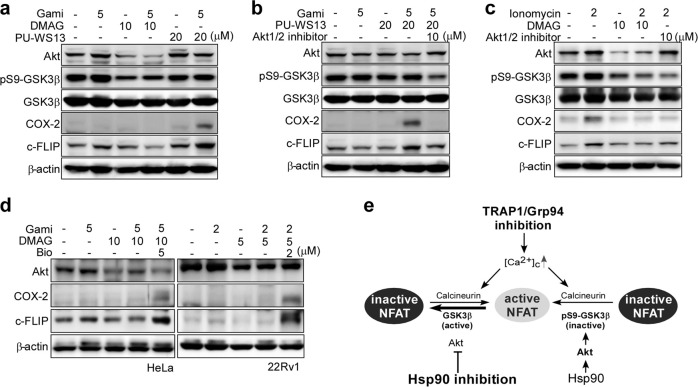
Akt inactivation blocks calcium/calcineurin-induced NFAT survival signals by activating GSK3β. **a** Decreased calcineurin/NFAT signaling upon degradation of Akt induced by Hsp90 inhibition. HeLa cells were incubated for 6 h with drugs and then analyzed by western blotting. **b** Decreased calcineurin/NFAT signaling upon Akt inactivation. HeLa cells were incubated for 6 h with drugs and then analyzed by western blotting. **c** Ionomycin induces calcium-mediated NFAT signaling. HeLa cells were incubated for 6 h with drugs and then analyzed by western blotting. **d** GSK3β-mediated NFAT signaling in cancer cells after combined drug treatment. HeLa and 22Rv1 cells were incubated for 6 h with drugs and then analyzed by western blotting. **e** Overview of Hsp90 paralog inactivation and NFAT signaling.

Calcineurin dephosphorylates and activates NFAT by triggering its nuclear translocation; this may have protumorigenic effects by supporting the proliferation and survival of cancer cells^44^. Thus, we examined whether activated calcineurin increases the transcriptional activity of NFAT after combined drug treatment. As reported previously, ionomycin-induced increases in cytoplasmic calcium were sufficient to trigger expression of the NFAT target genes COX-2 and c-FLIP in HeLa cells (Supplementary Fig. 5a)^45–47^. Similarly, NFAT activation was observed in HeLa cells after inhibition of Grp94 and TRAP1 (Fig. 5a). However, pan-Hsp90 inhibition via combined treatment with DMAG and gamitrinib did not increase the expression of COX-2 and c-FLIP (Fig. 5a). The data strongly suggested that Hsp90 participates in the NFAT activation pathway, probably in an indirect manner, by regulating the activities of its client proteins. Since Akt is an Hsp90 client protein that inactivates glycogen synthase kinase 3β (GSK3β) by phosphorylating residue Ser9^48^, we speculated that Akt suppressed the activity of GSK3β (an antagonist of NFAT) to maintain active NFAT. Consistently, expression of the inactive phosphor-form of GSK3β was reduced after inhibition of all Hsp90 family proteins (DMAG + Gami; Fig. 5a, b) or Hsp90 alone (DMAG; Fig. 5a) but was not reduced in the absence of Hsp90 inhibition (Gami, PU-WS13, or PU-WS13 + Gami; Fig. 5a). The data strongly indicate that pan-Hsp90 inhibition, due to the degradation of Akt, activates GSK3β and subsequently inhibits NFAT. Likewise, degradation or inhibition of Akt was closely associated with a reduction in the level of phosphor-GSK3β and was sufficient to reduce the expression of NFAT target genes (Fig. 5b, c). Additionally, treatment with the GSK3β inhibitor Bio^49^ fully reversed NFAT inactivation caused by pan-Hsp90 inhibition (Fig. 5d). The cytotoxic activity of the DMAG–gamitrinib combination was higher than that of the PU-WS13-gamitrinib combination (Supplementary Fig. 5b), and the cytotoxic activity of the PU-WS13-gamitrinib combination was dramatically improved when cells were cotreated with an Akt1/2 inhibitor (Supplementary Fig. 5c), suggesting that Akt inactivation is important for improving the anticancer activity of pan-Hsp90 inhibition. Taken together, the data argue that the synergistic cytotoxic effects resulting from simultaneous inactivation of Hsp90, Grp94, and TRAP1 are due to impaired calcium homeostasis and disruption of Hsp90 client protein networks in cancer cells (Fig. 5e).

### Combined drug treatment promotes mitochondrial dysfunction

Cytoplasmic calcium regulates a variety of cellular processes, including mitochondrial fusion/fission dynamics, which is closely related to the control of cell death pathways^50,51^. Combined treatment with gamitrinib and DMAG induced mitochondrial fragmentation to a greater extent than single agent treatment; however, this was reversed by the calcium chelator BAPTA-2AM (Supplementary Fig. 6a), suggesting calcium-induced mitochondrial fragmentation. Calcineurin dephosphorylates dynamin-related protein 1 (Drp1), a central regulator of the mitochondrial fission process, resulting in its translocation to the mitochondria and subsequent mitochondrial fission^52^. Consistent with this, combined drug treatment increased the accumulation of Drp1 in the mitochondria (Supplementary Fig. 6b); treatment with a Drp1-specific inhibitor (Mdivi-1)^53^ and siRNA reduced the effects of combined drug treatment on mitochondrial fragmentation (Supplementary Fig. 6c, d). Mitochondrial translocation of Drp1 triggers apoptosis via mitochondrial recruitment of Bax^54^. Likewise, treatment with the Drp1 inhibitor Mdivi-1 reduced mitochondrial recruitment of Bax and subsequent discharge of cytochrome c after combined drug treatment (Supplementary Fig. 6e). Inhibiting calcineurin using siRNAs reduced the mitochondrial fragmentation induced by combined drug treatment (Supplementary Fig. 6f), suggesting that activated calcineurin is responsible for mitochondrial recruitment of Drp1. Furthermore, the cytotoxic effects of combined drug treatment fell significantly after treatment with Mdivi-1, FK506, or BAPTA (Supplementary Fig. 6g). Taken together, these data suggest that activation of calcineurin by combined drug treatment promotes mitochondrial fragmentation by recruiting Drp1 to the mitochondria to further augment cytotoxicity against cancer cells.

### DN401 inhibits all Hsp90 paralogs simultaneously in vivo

**Fig. 6:**
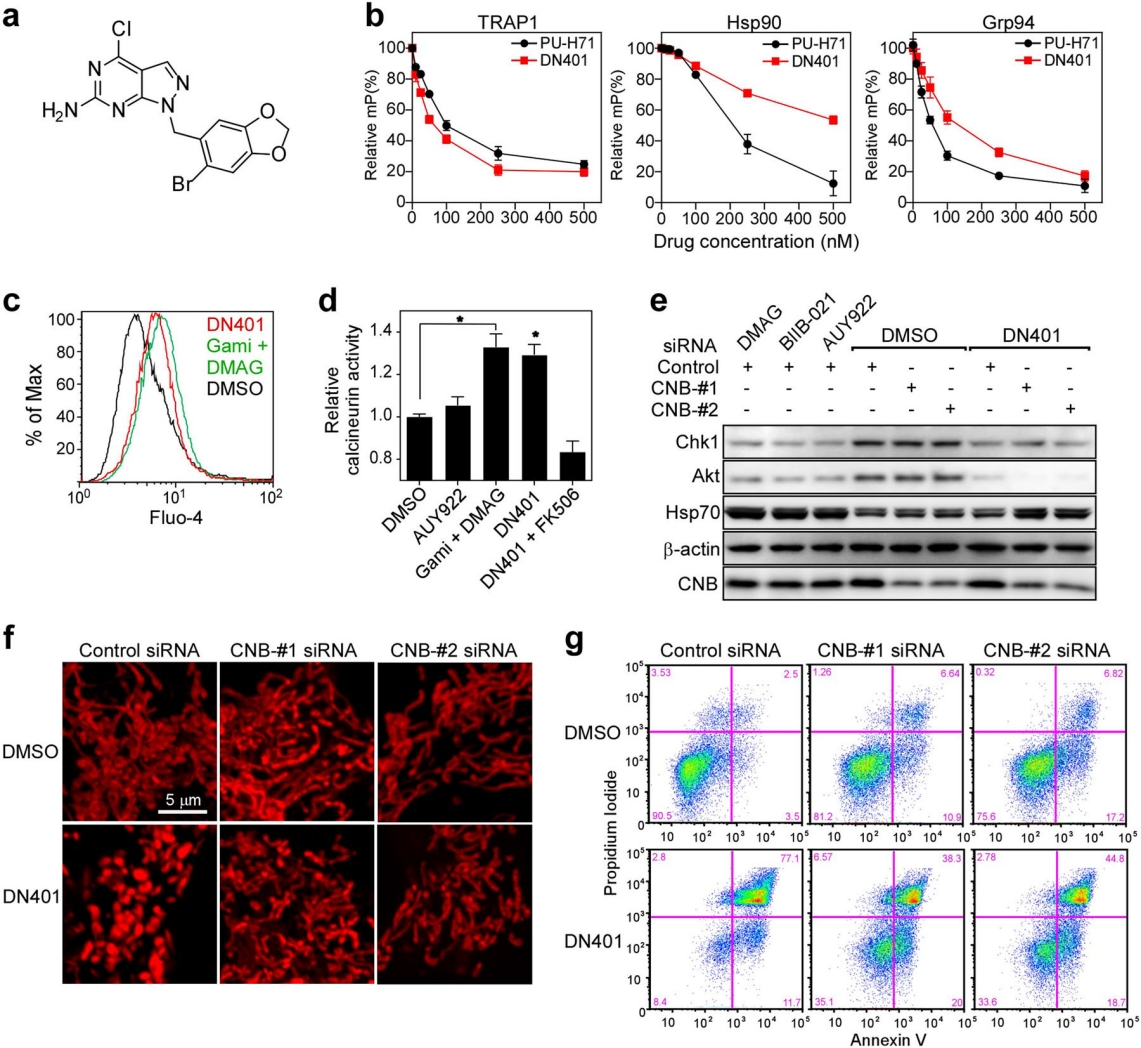
DN401 is a potent pan-inhibitor of Hsp90 family proteins, resulting in increased calcineurin activity. **a** Structure of DN401. **b** Binding affinity of DN401 for full-length TRAP1, Hsp90, and GST-Grp94. Fluorescence polarization was measured using purified recombinant proteins and PU-H71-FITC3. The results were compared with maximum and minimum millipolarization (mP) values and presented as the mean ± SEM of two independent experiments. **c** Fluo-4 AM staining. HeLa cells were treated for 6 h with 10 μM gamitrinib, 20 μM DMAG, and 20 μM DN401. Fluo-4 AM-loaded HeLa cells were analyzed by flow cytometry (BD FACSCalibur™) by gating on living cells. **d** Calcineurin phosphatase activity. HeLa cells were treated for 6 h with 10 μM AUY922, 5 μM gamitrinib, 10 μM DMAG, 10 μM DN401, and 10 μM FK506. Calcineurin activity in total cell extracts was analyzed using calcineurin phosphatase assay kits. Activity is shown relative to that in DMSO-treated controls; data are presented as the mean ± SEM. **e** Effect of DN401 on HSF1 signaling. HeLa cells were treated for 24 h with control or CNB-#1 siRNA, incubated with drugs (10 μM) for 6 h and then analyzed by western blotting. **f** Calcineurin-mediated mitochondrial fragmentation. HeLa cells were treated for 24 h with control or calcineurin subunit B (CNB) siRNAs. MitoTracker-loaded HeLa cells were treated for 6 h with DMSO or 10 μM DN-401 and then analyzed by confocal microscopy. Bar, 5 μm. **g** Calcineurin-mediated cytotoxicity. HeLa cells were treated for 24 h with 10 μM DN401 in the presence of control, CNB-1, or CNB-2 siRNAs, stained with PI/Annexin V, and analyzed by flow cytometry (BD FACSCalibur™).

According to our data, the anticancer activity of current Hsp90 inhibitors can be improved if mitochondrial TRAP1 is inactivated. Thus, we analyzed Hsp90 inhibitors to examine their ability to inhibit mitochondrial TRAP1 and identified DN401^12^ as showing inhibitory activity against all paralogs in vivo (Fig. 6a). DN401 strongly inhibited TRAP1 and Grp94 and weakly inhibited Hsp90 in vitro (Fig. 6b). Similar to combined treatment with DMAG and gamitrinib, DN401 degraded client proteins of Hsp90 (Akt, Chk1, and HER2), Grp94 (Her2) and TRAP1 (SIRT3, Sorcin, and SDHB) without inducing Hsp70, suggesting that the drug is an in vivo pan-Hsp90 family protein inhibitor (Supplementary Fig. 7a). DN401 treatment increased cellular calcium concentrations and calcineurin activity (Fig. 6c, d); ablation of calcineurin by siRNA reversed the DN401-mediated suppression of Hsp70 (Fig. 6e). In addition, because Akt was degraded, DN401 did not increase c-FLIP, which is a target gene of NFAT signaling (Supplementary Fig. 7a). Furthermore, DN401 treatment increased mitochondrial fragmentation (Fig. 6f) and increased apoptotic cell death (Fig. 6g), which was reduced by knockdown of calcineurin (Fig. 6f, g and Supplementary Fig. 7b). Collectively, the data show that DN401 inhibits Hsp90, Grp94, and TRAP1 (which are localized in different cellular organelles) simultaneously in vivo, thereby increasing the anticancer activity of the drug via a mechanism similar to that underlying combination treatment with DMAG and gamitrinib.

## Discussion

Here, we showed that the anticancer activity of the current Hsp90 inhibitors can be improved by inhibiting mitochondrial TRAP1. Experimental data generated using genetic knockdown and specific inhibitors targeting individual Hsp90 paralogs indicate that simultaneous inactivation of all Hsp90 family proteins (Hsp90, Grp94, and TRAP1) induces not only individual cellular compartment-specific proteotoxic stresses but also increases cytoplasmic calcium in cancer cells. The calcium-activated protein phosphatase calcineurin completely suppressed the major adverse effects of Hsp90 inhibition (HSF1 activation). The pyrazolopyrimidine scaffold inhibitor DN401 inhibited all Hsp90 paralog proteins in vivo, thereby increasing anticancer activity.

Considering that client proteins of Hsp90 family proteins are involved in multiple tumorigenic pathways in different cellular compartments, simultaneous inactivation of all Hsp90 family proteins (pan-Hsp90 inhibition) is expected to show more potent anticancer activity than single paralog-specific inhibition. This occurs through disruption of multiple tumor-supporting pathways within each organelle. Our experimental data show that, as expected, pan-Hsp90 inhibition not only induced the degradation of client proteins in individual organelles but also discharged calcium from the ER and mitochondria due to the compromised functions of Grp94 and TRAP1. The discharged calcium played important roles in amplifying organelle-specific stresses through activation of calcineurin, which consequently suppressed prosurvival signals (HSF1 activation) and switched on additional cell death signals (translocation of mitochondrial Bax after Drp1 activation). Although calcineurin activates prosurvival NFAT, calcineurin activation after pan-Hsp90 inhibition did not activate NFAT because Hsp90 inhibition degraded its client protein Akt, which is required for NFAT activation. Thus, multiple inhibitory effects of pan-Hsp90 inhibition on spatially separated chaperones led to potent anticancer effects.

Our conclusions raise an important question that has been ignored during the development of Hsp90 inhibitors but can affect both the mechanism of drug action and the anticancer activity of drugs markedly: are Hsp90 inhibitors capable of inactivating all Hsp90 family proteins within different cellular compartments of cancer cells? According to our experimental data^13,31^, the current Hsp90 inhibitors are unable to inhibit the mitochondrial pool of Hsp90 (TRAP1) in vivo due to inefficient drug accumulation inside mitochondria. It is very evident that Hsp90 inhibitors with TRAP1 inhibitory activity in vivo will show stronger anticancer activity than those without TRAP1 inhibitory activity. The next important question is as follows: is it possible to develop real pan-Hsp90 inhibitors that function in vivo? Here, we showed that DN401 is indeed a pan-Hsp90 inhibitor with potent anticancer activity in vivo. Since DN401 is a derivative of the well-known purine scaffold Hsp90 inhibitors PU-H71 and BIIB-021^12^, it is not only a proof-of-concept pan-Hsp90 inhibitor but also provides experimental evidence that the development of such drugs is feasible.

Taken together, our data clearly show that simultaneous inactivation of Hsp90 paralogs is cytotoxic to cancer cells without inducing protective mechanisms such as Hsp70 expression. Therefore, increasing the in vivo TRAP1-inhibiting activity of current Hsp90 inhibitors is a feasible and effective strategy for developing potent anticancer therapeutics.

## Supplementary information

supplementary information
