# Supplementary material for: Unleashing the full potential of Hsp90 inhibitors as cancer therapeutics through simultaneous inactivation of Hsp90, Grp94, and TRAP1
Source: Exp Mol Med. 2020 Jan 20;52(1):79–91. doi: 10.1038/s12276-019-0360-x (PMC7000702; doi:10.1038/s12276-019-0360-x)
Supplement: Supplementary file 1 — supplementary information [file 12276_2019_360_MOESM1_ESM.docx]

**Supplementary table 1. Combination index (CI) of the drug combination in cancer cells**

| Cell line | origin | drug ratio | CI at ED_50_ | CI at ED_75_ | CI at ED_90_ |
| --- | --- | --- | --- | --- | --- |
|  |  | Gami : DMAG |  |  |  |
| HeLa | cervix | 1 : 2 | 0.33±0.03 | 0.41±0.04 | 0.5±0.14 |
| 22Rv1 | prostate | 1 : 2 | 0.43±0.09 | 0.46±0.02 | 0.52±0.14 |
| A172 | brain | 1 : 1 | 0.42±0.01 | 0.36±0.26 | 0.63±0.11 |
| ACHN | kidney | 2 : 1 | 0.05±0.06 | 0.09±0.11 | 0.22±0.16 |
| NCI-H460 | lung | 1 : 2 | 0.39±0.05 | 0.47±0.07 | 0.57±0.12 |
| SK-Hep1 | liver | 5 : 1 | 0.73±0.52 | 0.71±0.29 | 0.74±0.26 |

NOTE: Cancer cells were treated with various concentrations of drugs at a fixed ratio, as indicated. CI values at 50% (ED50) and 75% effective doses (ED75) were calculated from isobologram analyses. Data are expressed as the mean±1.96 s.d. (95% confidence interval) of two independent experiments, each performed in triplicate.

**Supplementary figures**


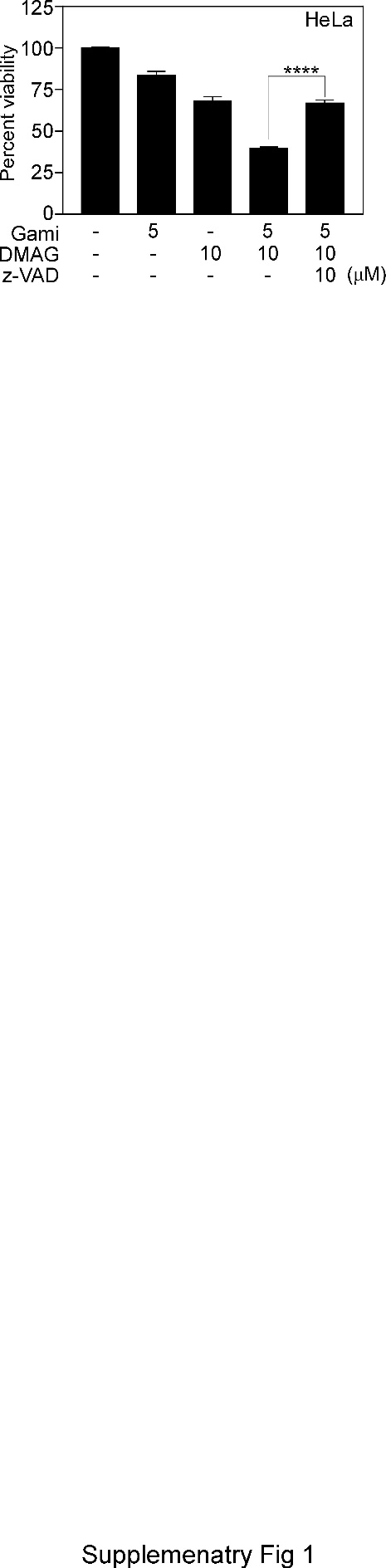


**Supplementary Fig. 1. Apoptosis induced by drug combinations**.

HeLa cells were treated for 24 h with drugs as indicated and cell viability was analyzed in an MTT assay.


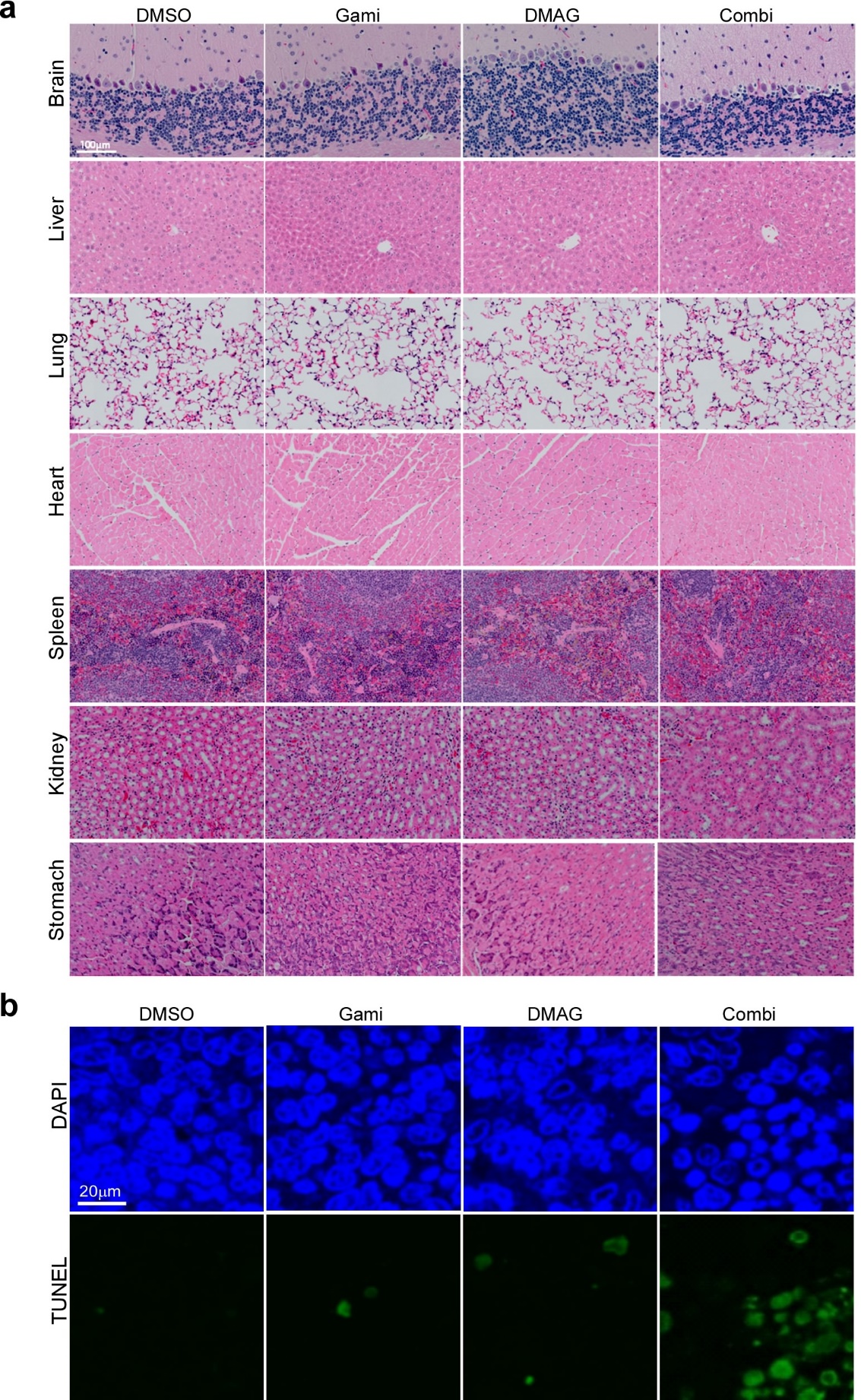


**Supplementary Fig. 2. Effect of combination drug treatment on normal tissues and tumor tissues.**

(a) Hematoxylin and eosin staining. Animals were sacrificed at the end of the experiment and organs were collected, fixed, stained, sectioned, and analyzed under a light microscope. Bar, 100 µm. (b) Analysis of apoptotic cell death. Tumors from sacrificed mice were collected, fixed, sectioned, and analyzed in a TUNEL assay (Roche). Bar, 20 µm.

**
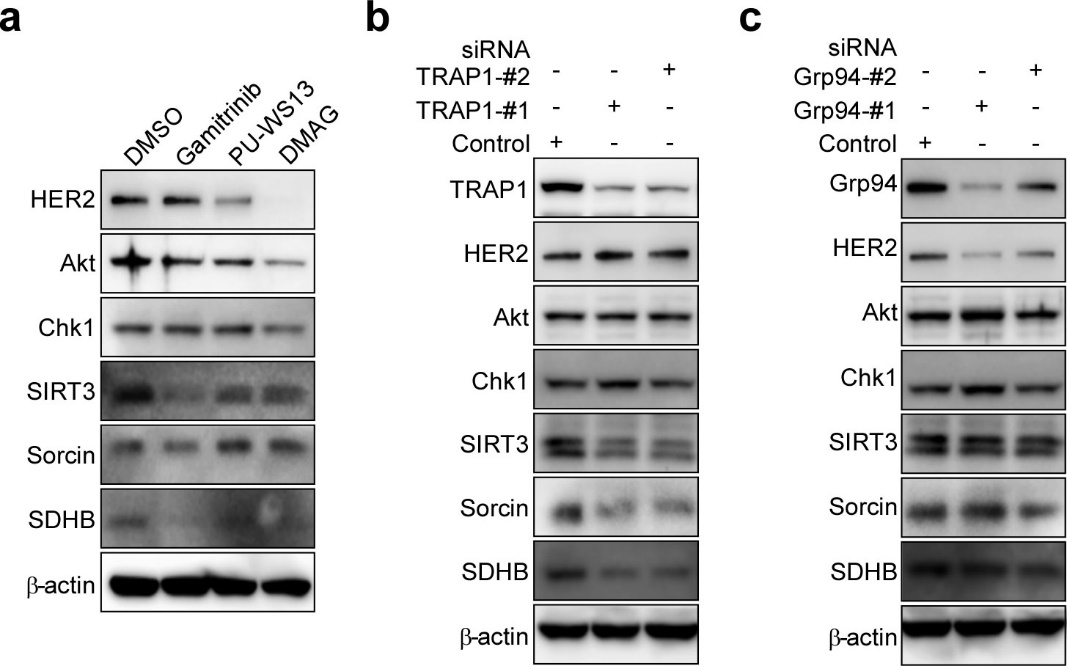
**

**Supplementary Fig. 3. Degradation of client protein upon inhibition of an Hsp90 paralog**.

(a) Degradation of a client protein on inhibition of an Hsp90 paralog. HeLa cells were treated with 5 μM Gamitrinib, 20 μM PU-WS13, and 10 μM DMAG as indicated for 6 h and analyzed by western blotting. (b) Client proteins of TRAP1. Control or TRAP1 siRNA-transfected HeLa cells were analyzed by western blotting. (c) Client proteins of Grp94. Control or Grp94 siRNA-transfected HeLa cells were analyzed by western blotting.


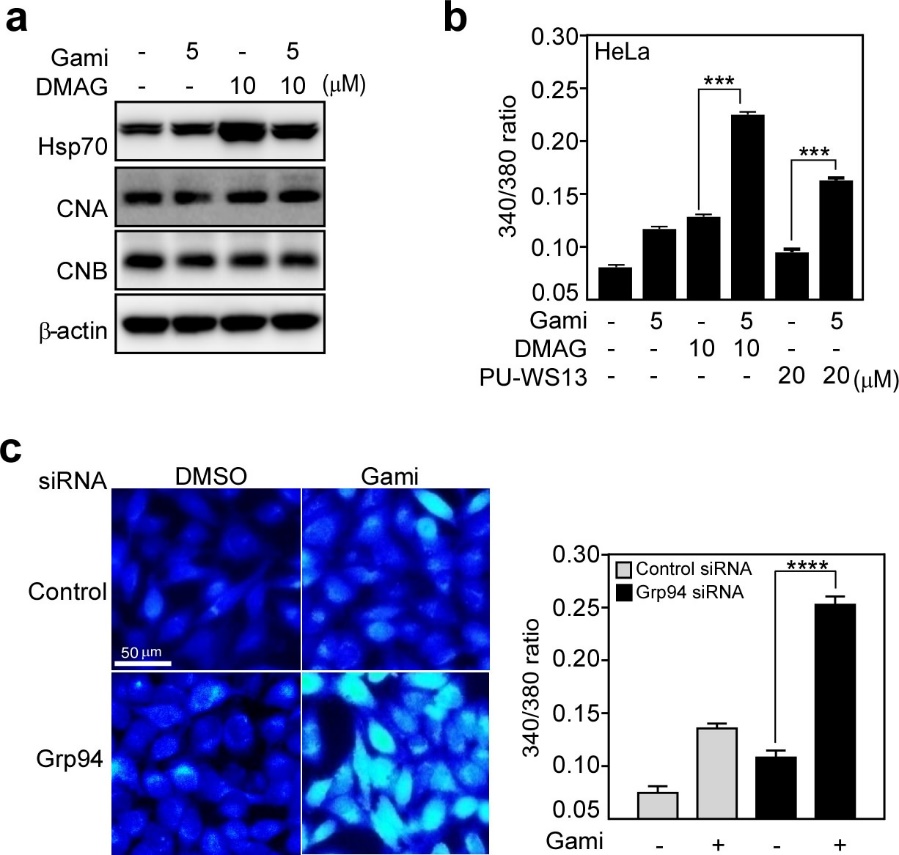


**Supplementary Fig. 4. Cytosolic calcium release upon combination drug treatment.**

(a) Calcineurin expression upon combination drug treatment. HeLa cells were treated with drugs for 6 h and analyzed by western blotting. (b) Cytoplasmic calcium release after drug treatment. HeLa cells were stained with Fura-2AM after exposure to drugs for 6 h and analyzed under an IX81 ZDC microscope (Olympus). Images showing the 340/380 fluorescence ratio, as analyzed by the Xcellence software package (Olympus). (c) Fura-2AM staining after Grp94 knockdown. Control or Grp94 siRNA-transfected HeLa cells were treated for 6 h with DMSO or 5 μM gamitrinib and analyzed under an IX81 ZDC microscope (Olympus) using the Xcellence software package (Olympus). A representative image (left) and the Fluorescence ratio (340/380, right) are shown in the bar graph. Data are expressed as the mean ± SEM. Data were collected from 40 cells in two independent experiments. Bar, 50 µm. *** *p* <0.001, *****p* <0.0001.


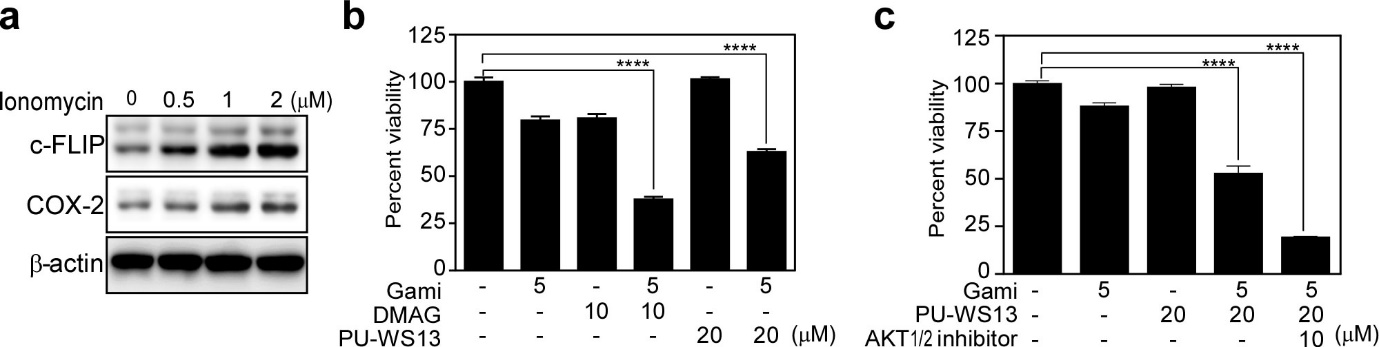


**Supplementary Fig. 5. Calcium-induced NFAT signaling upon combination drug treatment.**

(a) Calcium-induced NFAT signaling. 22Rv1 cells were treated with ionomycin for 6 h and proteins encoded by NFAT target genes were analyzed by western blotting. (b) Cytotoxicity induced by combination drug treatment. HeLa cells were treated with drugs for 24 h and cell viability measured in an MTT assay. (c) Cytotoxicity induced by combination treatment with an AKT inhibitor. HeLa cells were treated with drug for 24 h and cell viability measured in an MTT assay. In (b) and (c), data are expressed as the mean ± SEM of two independent experiments. *****p* <0.0001.


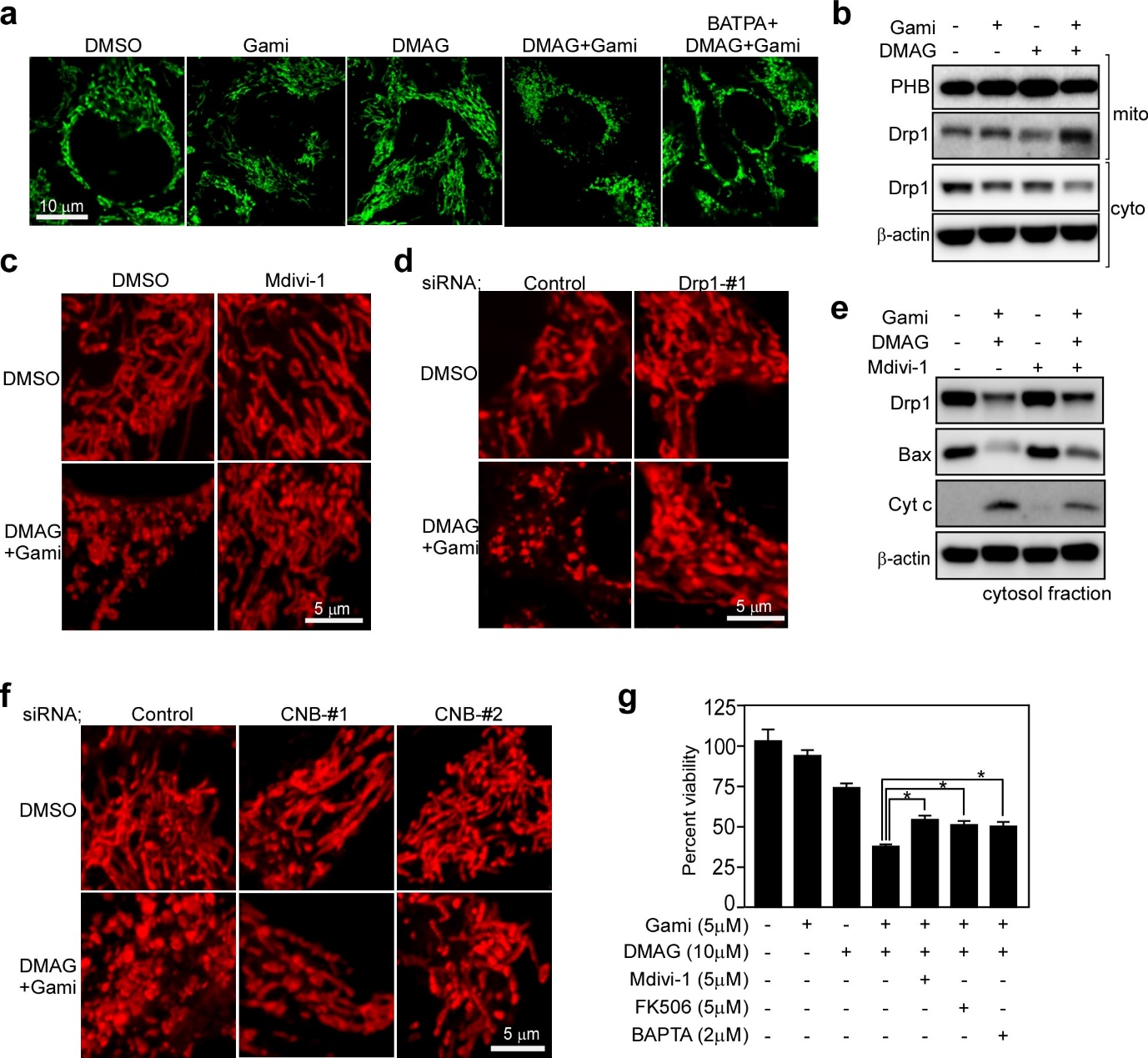


**Supplementary Fig. 6. Mitochondrial dysfunction induced by combination drug treatment.**

(a) Calcium-induced mitochondrial fragmentation. MitoTracker-labeled HeLa cells were treated with 5 μM gamitrinib, 10 μM DMAG, and 10 μM BAPTA-2AM for 6 h and analyzed under a confocal microscope. Bar, 10 µm. (b) Mitochondrial recruitment of Drp1. HeLa cells were treated with 5 μM gamitrinib and/or 10 μM DMAG for 6 h and the isolated mitochondrial and cytosolic fractions were analyzed by western blotting. (c) Drp1-mediated mitochondrial fragmentation. MitoTracker-labeled HeLa cells were treated with 5 μM gamitrinib, 10 μM DMAG and/or 10 μM Mdivi-1 for 6 h and analyzed under a confocal microscope. Bar, 5 µm. (d) Drp1 silencing. HeLa cells were treated with siRNA control or siRNA Drp1 for 24 h, followed by 5 μM gamitrinib or 10 μM DMAG for 6 h. Next, MitoTracker-labeled HeLa cells were analyzed under a confocal microscope. Bar, 5 µm. (e) Drp1-mediated release of cytochrome c. HeLa cells were treated with 5 μM gamitrinib, 10 μM DMAG, and/or 10 μM Mdivi-1 for 6 h and the isolated cytosolic fraction was analyzed by western blotting. (f) Calcineurin-induced mitochondrial fragmentation. Control or CNB siRNA-transfected HeLa cells were treated with 5 μM gamitrinib and 10 μM DMAG for 6 h, stained with MitoTracker and analyzed under a confocal microscope. Bar, 5 µm. (g) Cytotoxicity induced by combination drug treatment. HeLa cells were treated with drugs for 24 h as indicated and cell viability measured in an MTT assay. **p* <0.05. Data are expressed as the mean ± SEM in two independent experiments.


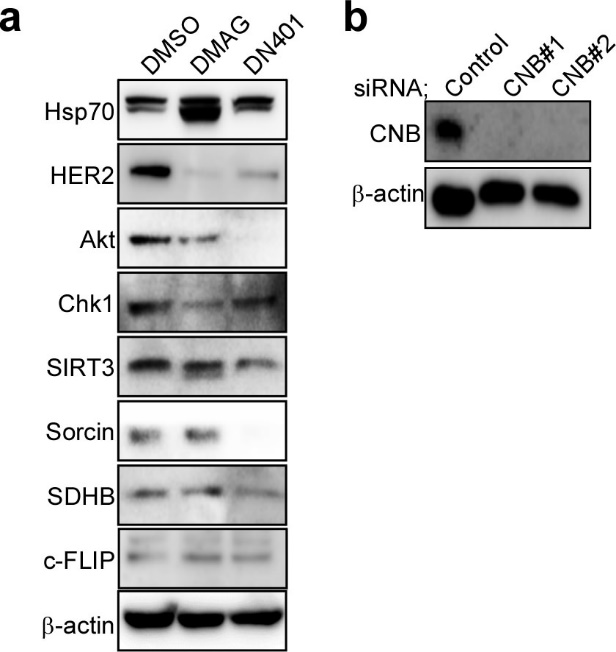


**Supplementary Fig. 7. Effect of a pan Hsp90 inhibitor, DN401, and silencing of CNB.**

(a) HeLa cells were treated with 10 μM DMAG and 10 μM DN401 for 6 h and analyzed by western blotting. (b) Efficiency of CNB silencing. HeLa cells were treated for 24 h with siRNA control or siRNA CNB#1 and CNB#2, and cell lysates analyzed by western blotting.
